# Main and interactive effects of physical activity, fitness and body mass in the prevention of cancer from the Copenhagen Male Study

**DOI:** 10.1038/s41598-018-30280-5

**Published:** 2018-08-06

**Authors:** Carlos Nunez, Johan Clausen, Magnus Thorsten Jensen, Andreas Holtermann, Finn Gyntelberg, Adrian Bauman

**Affiliations:** 1 Cancer Research Division, Cancer Council NSW, Sydney, Australia; 2 The University of Sydney, School of Public Health, Sydney, Australia; 3 National Research Centre for the Working Environment, Copenhagen, Denmark; 4 Department of Cardiology, Herlev-Gentofte Hospital, Hellerup, Denmark; 5 Epidemiological Research Unit, Departments of Occupational and Environmental Medicine, The Copenhagen Male Study, Bispebjerg University Hospital, Copenhagen, Denmark

## Abstract

Little knowledge exists about the role of cardiorespiratory fitness (CRF) or its interaction with excess adiposity determined by body mass index (BMI) in cancer prevention. A total of 5,128 middle-aged men, without a history of cancer at baseline in 1970–71, were examined for subsequent incidence and mortality of several cancer types. Participants’ data were linked with cancer registration and mortality data to March 2017. During 47 years of follow-up, a total of 1,920 incident cases and 1,638 cancer-related deaths were ascertained. BMI, particularly obesity, was associated with (i) incidence and (ii) mortality from respiratory/thoracic cancers; and (iii) all cancer-cause mortality. The respective adjusted hazard ratios (HRs) were: (i) 0.51 (95%CI:0.32–0.79), (ii) 0.48 (95%CI:0.30–0.75) and (iii) 0.73 (95%CI:0.59–0.89) when compared obese men (BMI ≥30 kg/m^2^) to men with healthy-BMI (<25 kg/m^2^). Increasing CRF was inversely associated with incidence and mortality of respiratory/thoracic cancers, HRs 0.78 (95%CI:0.67–0.90) and 0.73 (95%CI:0.63–0.84) respectively; and all cancer-cause incidence 0.92 (95%CI:0.86–0.98) and mortality 0.85 (95%CI:0.79–0.91). Physical activity (PA) was not associated with most outcomes. We found no evidence of interactions between CRF or PA and BMI on cancer risk. This evidence suggests that midlife CRF is associated with lowered risk of cancer incidence and mortality with no evidence of cancer risk modification by BMI.

## Introduction

Cancer is a prominent cause of morbidity and mortality worldwide, accounting for 14 million new cases and 8 million deaths in 2012^1^; of which, 7.4 million incident cases and 4.6 million cancer-related deaths occurred in men. Higher incidence rates were reported in OECD nations whereas higher mortality was documented in developing countries^1^. Globally, the highest incidence rate amongst men and women was reported in Denmark, age-standardised 338 per 100.000 people in 2012^2^, with 1 in 3 cancers being potentially preventable thought modification of lifestyle risk factors^3^.

Lifestyle modifications are promising strategies to reduce cancer risk. High body mass, defined as having a body mass index (BMI) of 25 kg/m^2^ or greater, may increase cancer risk resulting in higher incidence and death^4^. The increased prevalence of overweight and obesity has virtually spread to every country. In 2014, 69.2% of Danish males were either overweight or obese^5^; this rise in excess body mass is a major concern as there is sufficient evidence for an associated increased risk for 13 types of cancer, including esophageal adenocarcinoma, gastric cardia, colorectum, liver, gallbladder and pancreatic cancers^6^.

Conversely, physical activity (PA) has consistently been linked to a decreased risk of colon cancer, is probably associated with reduced risks of postmenopausal breast and endometrial cancer; and less consistent with other types of cancer^7^. This lack of association may be hampered due to imprecise measurement of this complex and multifaceted behaviour^8^, which is usually determined through self-report in most epidemiological studies^9^. Cardiorespiratory fitness (CRF), an objective attribute of repetitive training activities and of genetics, is measured by the maximal oxygen uptake (VO_2_ max) required by the body during sustained physical exertion^10^. Thus, CRF provides the most accurate population measure of regular fitness^11^. However, little is known about its impact on cancer prevention, as very few studies have examined this objective measurement of PA with the most common incident cancers in men^10,12–14^. Furthermore, the possible pre-diagnostic role of CRF in cancer specific mortality has not been fully explored^15^.

Although PA and high body mass are considered independent risk factors for some types of cancer^7,16^, the interaction between these two determinants and cancer outcomes has not been well investigated using objective measures of PA^10^. Disease risk modification has been documented in several epidemiological studies of all-cause mortality and cardio-metabolic outcomes; in those studies, obese individuals with high levels of PA or fitness had lower cardio-metabolic risk or better survival compared to inactive or unfit individuals with a healthy BMI^17^. This phenomenon is also known as “fat but fit”^18^. Nevertheless, cancer risk modification still remains to be investigated.

The current prospective study examined main and interactive effects of PA, fitness and BMI on the incidence and mortality of different cancer groups in the Copenhagen Male Study.

## Results

Of 5,245 participants included in the examination, 117 were excluded due to a history of cancer other than non-melanocytic skin cancer prior to recruitment (n = 78) or did not perform the exercise test (n = 39). After exclusions, a total of 5,128 participants remained for analysis. There were 391 incident cases of prostate cancer (PC), colorectal cancer (CRC) (n = 299), oral/digestive (n = 546), respiratory/intrathoracic (n = 455), genito-urinary (n = 571), other cancers (n = 348) and all-cancers combined (n = 1,920). Additionally, there were 253 deaths due to PC, CRC (n = 218), oral/digestive (n = 446), respiratory/intrathoracic (n = 482), genito-urinary (n = 380), other cancers (n = 322) and all-cancers combined (n = 1638). Mean follow-up was 29.7 years, ranging from 0.3 to 44.1 years. The mean age at recruitment was 48.8 years (age range 39.0 to 61.0 years).

Baseline socio-demographic and lifestyle characteristics are shown for BMI, PA and CRF in Table 1. Participants in the highest BMI category (≥30 kg/m^2^) compared to the lowest (<25 kg/m^2^), were more likely to be older, of higher social class, to report more units of alcohol per day, to have higher mean systolic and diastolic blood pressure; and less likely to be current smokers. Compared to participants who reported almost no PA, those who reported a lot were more likely to be of higher social class, less likely to drink large quantities of alcohol and to have lower mean diastolic blood pressure. Participants who had high fitness levels compared to those with low fitness, were more likely to be younger, current smokers and to consume more grams of tobacco, less likely to drink large amounts of alcohol and to have much lower mean systolic and diastolic blood pressure.

**Table 1:** Baseline characteristics of participants in the CMS according to BMI, self-reported PA and CRF.

Characteristic	BMI Kg/m^2^			Physical activity			Cardiorespiratory fitness ml/kg/min*		
	<25	≥25–<30	≥30	Almost nothing	Some	A lot	Low (15–29)	Moderate (30–35)	High (36–78)
	n = 2455 (%)	n = 2328 (%)	n = 336 (%)	n = 887 (%)	n = 3687 (%)	n = 554 (%)	n = 1736 (%)	n = 1733 (%)	n = 1659 (%)
**Birth cohort**									
1910s	714 (29.1)	793 (34.1)	129 (38.4)	274 (30.9)	1,187 (32.2)	180 (32.5)	755 (43.5)	548 (31.6)	338 (20.4)
1920s	1,542 (62.8)	1,404 (60.3)	196 (58.3)	548 (61.8)	2,262 (61.4)	335 (60.5)	927 (53.4)	1,061 (61.2)	1,157 (69.7)
1930s	199 (8.1)	131 (5.6)	11 (3.3)	65 (7.3)	238 (6.4)	39 (7.0)	54 (3.1)	124 (7.2)	164 (9.9)
**Socioeconomic Status**									
Low	497 (20.3)	333 (14.3)	23 (6.8)	179 (20.2)	641 (17.4)	35 (6.3)	268 (15.5)	285 (16.5)	302 (18.2)
Middle	766 (31.2)	600 (25.8)	75 (22.3)	289 (32.6)	1,044 (28.4)	112 (20.3)	508 (29.3)	484 (28.0)	453 (27.3)
High	1,189 (48.5)	1,392 (59.9)	238 (70.9)	419 (47.2)	1,997 (54.2)	406 (73.4)	956 (55.2)	962 (55.5)	904 (54.5)
**Smoking statu**s									
Never	194 (7.9)	214 (9.2)	34 (10.1)	76 (8.5)	307 (8.3)	61 (11.0)	147 (8.5)	148 (8.5)	149 (9.0)
Former	397 (16.2)	506 (21.7)	81 (24.1)	171 (19.3)	711 (19.3)	102 (18.4)	377 (21.7)	347 (20.0)	260 (15.7)
Current	1,864 (75.9)	1,608 (69.1)	221 (65.8)	640 (72.2)	2,669 (72.4)	391 (70.6)	1,212 (69.8)	1,238 (71.5)	1,250 (75.3)
Mean grams of tobacco per day (s.d)	14.7 (11.2)	13.6 (12.0)	12.4 (11.5)	14.7 (12.1)	14.0 (11.5)	13.4 (11.6)	13.3 (11.5)	13.9 (11.6)	15.1 (11.8)
**Alcohol (units/day)**									
2 or less	2,092 (85.2)	1,826 (78.4)	212 (63.1)	695 (78.4)	2,999 (81.3)	444 (80.2)	1,318 (75.9)	1,426 (82.3)	1,394 (84.1)
3–5	308 (12.5)	406 (17.5)	97 (28.9)	141 (15.9)	576 (15.6)	95 (17.1)	339 (19.5)	245 (14.1)	228 (13.7)
6 or more	55 (2.3)	96 (4.1)	27 (8.0)	51 (5.7)	112 (3.1)	15 (2.7)	79 (4.6)	62 (3.6)	37 (2.2)
**Diabetes**									
Yes	22 (0.9)	16 (0.7)	5 (1.5)	12 (1.4)	27 (0.7)	4 (0.7)	21 (1.2)	11 (0.6)	11 (0.7)
**Previous AMI**									
Yes	36 (1.5)	28 (1.2)	1 (0.3)	14 (1.6)	49 (1.3)	2 (0.4)	30 (1.7)	26 (1.5)	9 (0.5)
Mean Systolic BP (s.d)	131.8 (18.2)	137.0 (19.3)	144.8 (21.9)	135.8 (19.7)	135.0 (19.3)	133.9 (18.9)	142.1 (20.6)	134.3 (18.1)	128.5 (16.5)
Mean Diastolic BP (s.d)	80.5 (10.9)	84.9 (11.5)	90.4 (12.9)	84.4 (12.2)	83.1 (11.5)	82.0 (11.7)	86.6 (12.4)	82.9 (11.1)	79.8 (10.4)

(%) Correspond to column percent.

AMI: acute myocardial infarction. BP: blood pressure in mmHg. (s.d): Standard deviation.

*The cut-offs low, moderate and high were obtained from tertiles of the actual distribution of CRF in this cohort.

BMI was associated with incidence and mortality of respiratory/thoracic cancers and all cancer-cause mortality (Tables 2 and 3). Participants with a BMI ≥30 kg/m^2^ had a 49% and 52% decreased risk of being diagnosed and dying from respiratory/thoracic cancers respectively, when compared to participants with a BMI <25 kg/m^2^. Additionally, participants categorised as obese had a 27% risk reduction of all cancer-cause mortality compared to participants with a healthy range BMI. Sensitivity analyses excluding underweight participants from the healthy BMI category did not appreciably change any effects of relative risk of neither incidence nor mortality (Supplementary material).

**Table 2:** Adjusted hazard ratios (HR) and 95% confidence intervals (CI) for cancer incidence according to time-varying BMI and PA; and baseline CRF in the CMS.

Cancer type	BMI Kg/m^2^ HR (95%CI)*					Physical Activity HR (95%CI)*				CRF*	
	Events	<25	≥25–<30	≥30	P-value^A^	Almost nothing	Some	A lot	P-value^A^	10 ml/kg/min (VO_2_ max)	P-value^A^
Prostate	391	1.00	1.06 (0.85–1.31)	0.84 (0.53–1.32)	0.56	1.00	0.78 (0.57–1.08)	0.78 (0.56–1.09)	0.29	1.02 (0.88–1.19)	0.75
Colorectal	299	1.00	1.07 (0.84–1.37)	0.68 (0.40–1.15)	0.20	1.00	1.17 (0.79–1.73)	1.06 (0.70–1.59)	0.60	0.94 (0.79–1.12)	0.48
Oral and digestive	546	1.00	1.10 (0.91–1.33)	0.99 (0.71–1.39)	0.54	1.00	1.23 (0.93–1.63)	1.01 (0.74–1.36)	0.15	0.90 (0.79–1.03)	0.13
Respiratory and thoracic	455	1.00	0.87 (0.71–1.06)	**0.51 (0.32–0.79)**	0.01	1.00	0.92 (0.69–1.22)	0.81 (0.59–1.10)	0.31	**0.78 (0.67–0.90)**	0.001
Genito-urinary	571	1.00	1.05 (0.87–1.25)	0.85 (0.59–1.22)	0.48	1.00	0.83 (0.64–1.08)	0.77 (0.58–1.01)	0.15	1.05 (0.93–1.18)	0.47
Other cancer	348	1.00	0.98 (0.78–1.24)	1.06 (0.69–1.61)	0.94	1.00	1.17 (0.81–1.67)	1.05 (0.72–1.53)	0.55	0.93 (0.79–1.09)	0.35
All-cancers	1,920	1.00	1.00 (0.91–1.11)	0.83 (0.68–1.00)	0.12	1.00	1.01 (0.87–1.16)	0.88 (0.75–1.03)	0.10	**0.92 (0.86–0.98)**	0.01

*Multivariable model adjusted for: birth decades, smoking and grams of tobacco a day, alcohol, SES, systolic blood pressure, diastolic blood pressure, previous AMI, diabetes and the other study variables. (All covariates are time-dependent except for birth decade, previous AMI, SES and CRF).

^**A‘**^P-value’ for each variable corresponds to a test of whether all HRs = 1.

**Table 3:** Adjusted hazard ratios (HR) and 95% confidence intervals (CI) for cancer related death according to time-varying BMI and PA; and baseline CRF in the CMS.

Cancer type	Events	BMI Kg/m^2^ HR (95%CI)*				Physical Activity HR (95%CI)*				CRF*	
		<25	≥25–<30	≥30	P-value^A^	Almost nothing	Some	A lot	P-value^A^	10 ml/kg/min (VO_2_ max)	P-value^A^
Prostate	253	1.00	1.04 (0.79–1.36)	0.72 (0.40–1.28)	0.43	1.00	0.77 (0.51–1.15)	0.81 (0.53–1.22)	0.44	1.02 (0.85–1.23)	0.80
Colorectal	218	1.00	0.89 (0.67–1.18)	0.63 (0.34–1.15)	0.30	1.00	0.96 (0.63–1.48)	0.85 (0.54–1.34)	0.66	0.91 (0.74–1.11)	0.35
Oral and digestive	446	1.00	0.96 (0.78–1.18)	0.91 (0.63–1.31)	0.84	1.00	1.12 (0.83–1.51)	0.82 (0.59–1.13)	0.22	0.89 (0.77–1.03)	0.12
Respiratory and thoracic	482	1.00	0.92 (0.75–1.11)	**0.48** (**0.30–0.75)**	0.005	1.00	0.95 (0.72–1.25)	0.81 (0.60–1.09)	0.23	**0.73** (**0.63–0.84)**	<0.001
Genito-urinary	380	1.00	0.97 (0.78–1.20)	0.72 (0.45–1.15)	0.38	1.00	0.98 (0.69–1.40)	0.99 (0.69–1.41)	0.99	0.97 (0.83–1.13)	0.67
Other cancer	322	1.00	0.78 (0.61–0.99)	0.75 (0.47–1.18)	0.10	1.00	1.27 (0.86–1.88)	1.16 (0.77–1.75)	0.44	0.86 (0.73–1.01)	0.07
All-cancers	1,638	1.00	0.91 (0.82–1.02)	**0.73** (**0.59–0.89)**	0.007	1.00	1.00 (0.86–1.17)	0.88 (0.75–1.04)	0.13	**0.85** (**0.79–0.91)**	<0.001

*Multivariable model adjusted for: birth decades, smoking and grams of tobacco a day, alcohol, SES, systolic blood pressure, diastolic blood pressure, previous AMI, diabetes and the other study variables. (All covariates are time-dependent except for birth decade, previous AMI, SES and CRF).

^**A‘**^P-value’ for each variable corresponds to a test of whether all HRs = 1.

PA was only related to genitourinary cancer incidence in sensitivity analysis; those participants who reported a lot of PA had a 25% decreased risk. The respective HR was 0.75 (95%CI: 0.57–0.99). Additionally, CRF was associated with reduced incidence and mortality of respiratory/thoracic cancers; and all cancers combined (Tables 2–3). Lower risks were noted for developing respiratory/thoracic cancers; and all-cancers combined per 10 ml/kg/min increase in VO_2_ max; the respective HRs were 0.78 (95%CI: 0.67–0.90) and 0.92 (95%CI:0.86–0.98). Besides, a 10 ml/kg/min increase in VO_2_ max was associated with a 27% and 15% decreased risk of respiratory and thoracic cancer mortality; and all cancer-cause mortality. Sensitivity analysis excluding the first ten years of follow-up did not substantially change any effects of relative risks for the different study variables (Supplementary Material).

**Figure 1:**
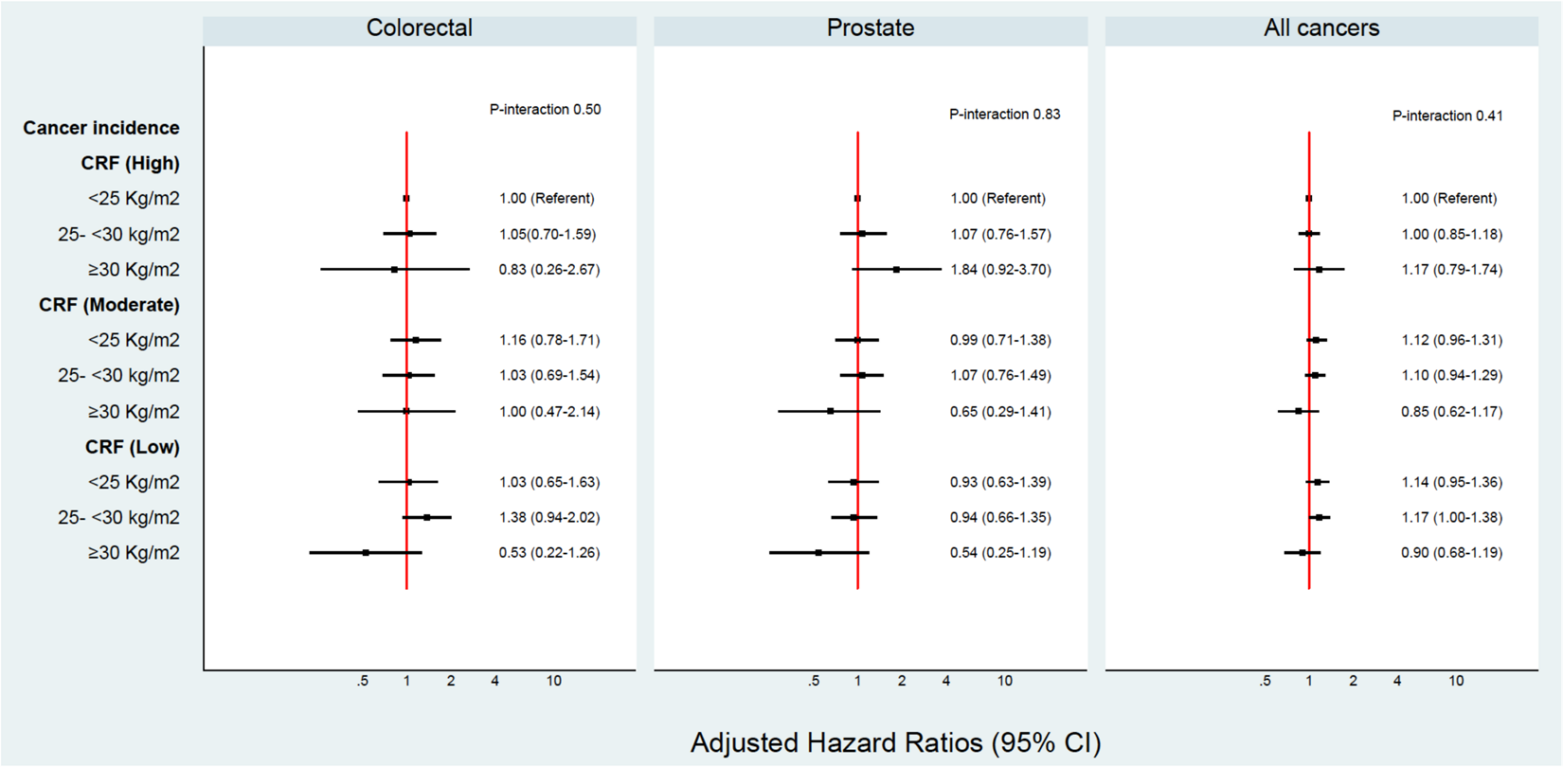
Multivariable model adjusted for: birth decades, smoking and grams of tobacco a day, alcohol, SES, systolic blood pressure, diastolic blood pressure, previous AMI, diabetes, physical activity, BMI, CRF and interaction between BMI-CRF.

**Figure 2:**
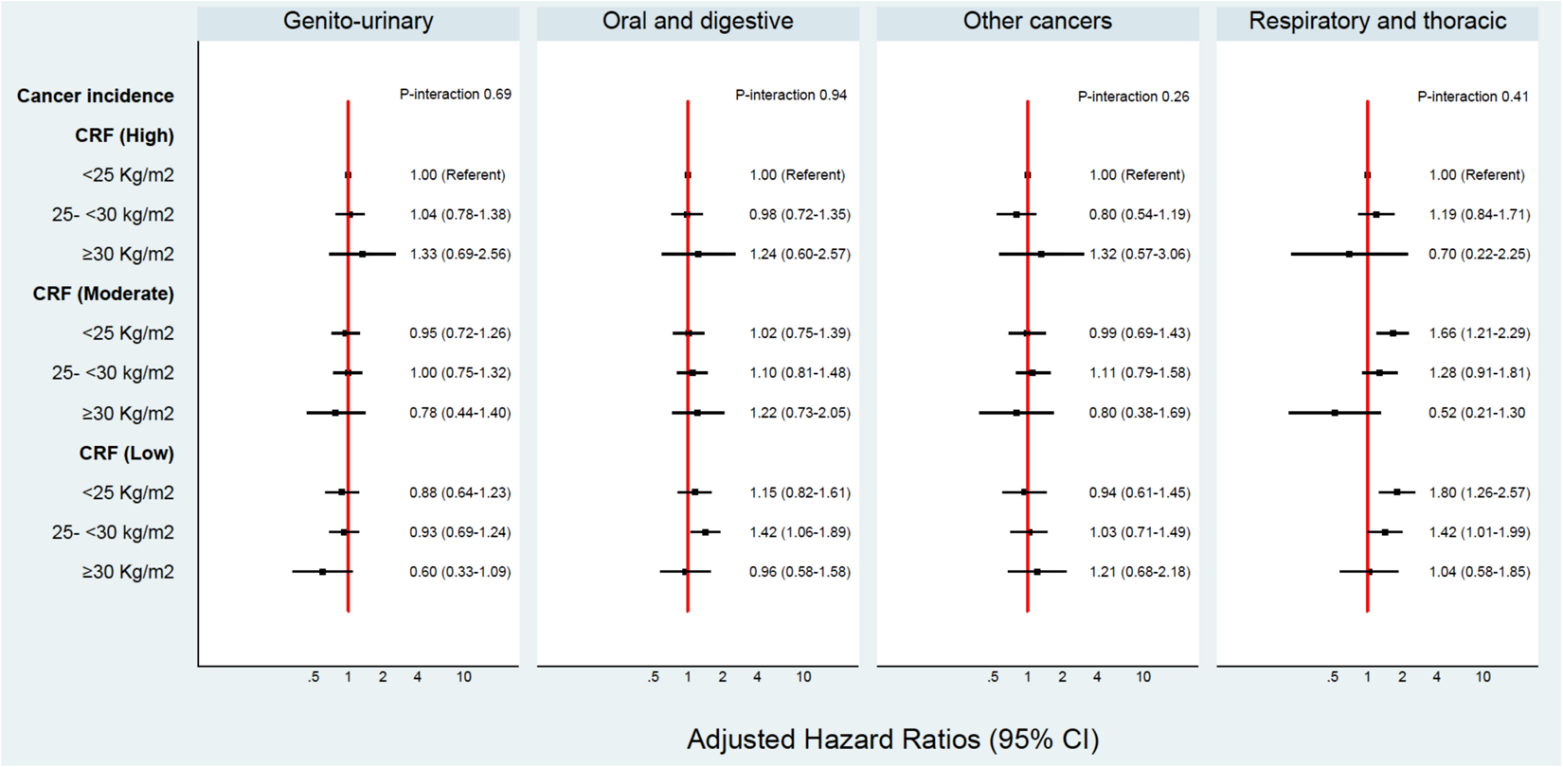
Multivariable model adjusted for: birth decades, smoking and grams of tobacco a day, alcohol, SES, systolic blood pressure, diastolic blood pressure, previous AMI, diabetes, physical activity, BMI, CRF and interaction between BMI-CRF.

**Figure 3:**
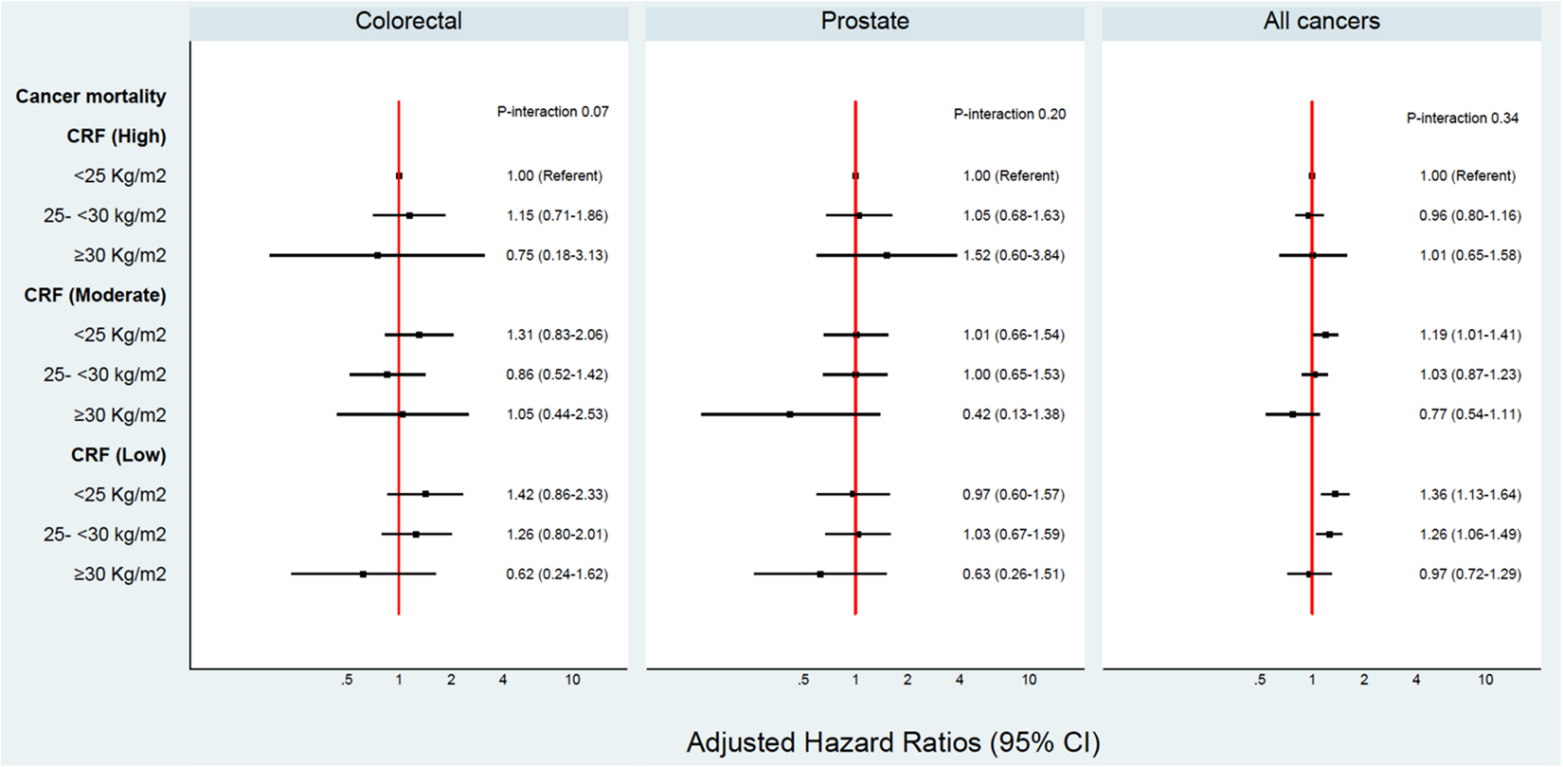
Multivariable model adjusted for: birth decades, smoking and grams of tobacco a day, alcohol, SES, systolic blood pressure, diastolic blood pressure, previous AMI, diabetes, physical activity, BMI, CRF and interaction between BMI-CRF.

**Figure 4:**
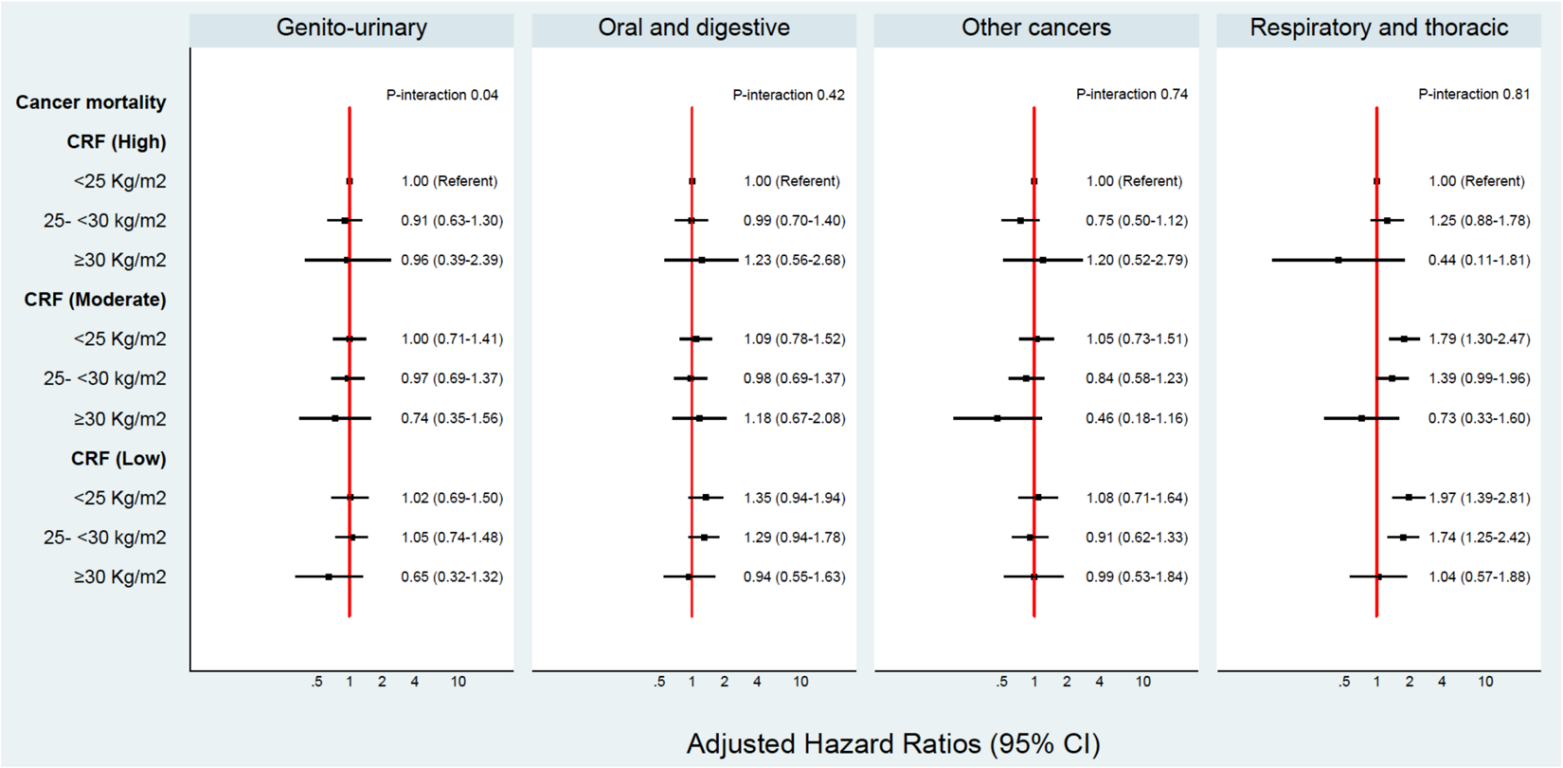
Multivariable model adjusted for: birth decades, smoking and grams of tobacco a day, alcohol, SES, systolic blood pressure, diastolic blood pressure, previous AMI, diabetes, physical activity, BMI, CRF and interaction between BMI-CRF.

In this cohort, 2.1% or 35 individuals were identified to be “fat but fit”, based on a BMI ≥ 30 kg/m^2^ and a high CRF (36–78 ml/kg/min). A p-value below 0.1 may be an acceptable cut-off for interactions^19^. Overall, no significant interactions were evident between BMI and CRF or BMI and PA on cancer risk. Figures 1, 2, 3 and 4 show the interactions between BMI-CRF and the interactions between BMI-PA are portrayed in the Supplementary material.

## Discussion

In this Danish cohort, BMI and CRF were independent predictors of cancer risk in men. Reduced risks of respiratory/thoracic cancer incidence and mortality; and all cancer-cause mortality were noticed for participants with BMI ≥30 kg/m^2^ compared to participants with a healthy BMI (<25 kg/m^2^). Increasing levels of CRF also lowered cancer risk incidence and mortality of respiratory/thoracic cancers; and all-cancers combined. Self-reported PA was not associated with most outcomes assessed. Additionally, we detected no evidence of interaction between the effects of BMI and PA or CRF on cancer risk. Testing the “fat but fit” concept was difficult in this cohort because obesity was not associated with higher risks of cancer. Obesity is usually a marker of social inequality in developed countries and these disparities are known to influence the burden of cancer^20^. However, in this early epidemiological study, obese participants were from high socioeconomic status (SES), and thus they might have different health-risk behaviour profiles, which may explain the reduced risk associated with obesity^20^. Additionally, confounding by smoking (smokers were thinner) could be another possible reason for the reduced risk observed for obesity^21^.

The International Agency for Research on Cancer (IARC) working group on body mass reported that there is sufficient evidence for a positive association between obesity and some gastrointestinal cancers, with the highest risk for esophageal adenocarcinoma and the lowest for colorectum or gallbladder^6^. Additionally, this group reported limited evidence for fatal PC and inadequate evidence for lung cancer (LC)^6^. A reduced risk of LC has been reported with obesity in a fairly recent systematic review and meta-analysis^21^. In our study, we also observed that obesity conferred lower risk of respiratory/thoracic cancers. Possible explanations for this obesity paradox include (i) confounding by smoking status since this behaviour influences body weight and body composition; and (ii) storage, mobilisation, and metabolism of carcinogen-DNA adducts by the adipose tissue^21^.

Ambivalence still persists in the association of BMI with cancer mortality^22^. Most studies on cancer-related death have observed an increased risk from all-cancer combined or CRC in men with obesity compared to their healthy BMI counterparts^22,23^. The elevated risk for all-cancers combined has been documented around 10% and 32% for CRC^22^. In a recent meta-analysis of nearly 4 million participants from different world regions, a BMI of 25 kg/m^2^ or greater was associated with increased all-cancer cause mortality in a log-linear manner which did not differ across each region^24^. The increased risk of cancer death was 10% for overweight, 31% for obesity grade I (BMI ≥30 to <35 kg/m^2^), 57% for obesity grade II (BMI ≥35 to <40 kg/m^2^) and 96% for obesity grade III (BMI ≥40 to <60 kg/m^2^)^24^. Mortality relationships with PC or other types of cancer are less consistent with conflicting results^22,23,25,26^.

Considerable evidence exists for a protective role of PA in lowering the risk of some types of cancer, particularly with colon cancer (CC)^27^. The World Cancer Research Fund (WCRF) and IARC have described the association between PA and LC as suggestive^7^. Nevertheless, a recent review of the literature suggests that the majority of studies support a role of PA in reducing LC incidence by 20–50% in men^28^; and proposes a weak inverse association of PA and PC with an average risk reduction of 10%^29^. Studies assessing CRF and cancer incidence have reported lower risks of LC or CC in participants with high levels of CRF compared to the lowest; the respective reduced risk were 55% and 44%^12,14^. However, the association with PC has produced inconsistent findings; an earlier analysis from an American cohort study reported a reduction of 74%^30^ while subsequent analysis observed an increased risk which ranges from 22% to 74%^10,14^. Our data suggest that increasing CRF levels lower cancer risk incidence of respiratory/thoracic cancers; and all-cancers combined.

Pre-diagnostic CRF was associated with a decreased risk of respiratory/thoracic cancer mortality; and all cancer-cause mortality even after adjusting for BMI levels; this shows that the apparent protection is not explain by adiposity as was previously suggested for several types of cancer^7^. We did not find evidence for a beneficial effect of PA or fitness on mortality for other clinical cancer groups. Genetics and habitual PA behaviour are considered the main determinants of CRF. Although the reported correlation between PA and CRF ranges from 60 to 70%^31^, this study suggests that CRF may be a better predictor of regular vigorous activity than subjective self-report measures on cancer outcomes. Additionally, PA captured by self-report was broad-ranging and the generic question used in this analysis may have introduced measurement error mis-classification, attenuating the observed association with cancer outcomes^32^.

In 2011, cancer as a single entity was the leading cause of death globally and 20 million new cancer cases are projected by 2025^1^. Therefore, the elucidation of the interaction between body mass and levels of PA or CRF on cancer outcomes is of public health interest because these independent lifestyle factors contribute independently to the burden of cancer^1^. Very few studies have examined this interaction on cancer incidence, providing contradictory results^10,33–38^. Four observational studies have focused on CC^33–36^; of them, only two case-control studies detected a significant interaction, reporting that high levels of PA offset risks among those with the highest BMI^35,36^. Two studies appraised this interaction on LC risk^37,38^; of which, a case-control study reported a significant interaction^37^. The authors noticed lower risk in healthy BMI or overweight individuals with high levels of activity but the same observation was not detected in obese individuals. Regarding prostate cancer, one prospective study noted a significant interaction where obese individuals with moderate or high levels of CRF offset obesity risks^10^. Case-control studies were more likely to report significant interactions than other epidemiological designs. To our knowledge, the interaction between BMI and PA on all cancer mortality has been assessed in one study, finding no significant interaction^39^. We found no evidence of interaction between BMI and levels of PA or CRF on cancer incidence or mortality. Despite yielding significant p-values for CRC and genito-urinary cancer mortality, stratified effect sizes were not significant. These spurious statistical interactions may have emerged from categorization of CRF and BMI in accordance with established values, producing unequal observations across different stratum or inappropriate median splits^40,41^.

This analysis has several strengths and limitations. Among its strengths are the prospective nature of the study design, the linkages of questionnaire data to deaths records and cancer registry; the long follow-up period, which was sufficient to allow the ascertainment of a large number of cancer end points and the objective assessment of physical fitness (CRF) which is not usually feasible in large studies. A limitation is that changes in health-related fitness could not be assessed since CRF was only collected at baseline. Additionally, CRF was estimated using an indirect method of VO_2_ max. However, this measurement is known to have a high correlation of 0.87 compared to more direct methods of estimating VO_2_ max^42^. Finally, confidence intervals of two-way interactions were wide within strata, perhaps suggesting limited statistical power to detect such interactions^19^.

In conclusion, findings from this study underline the importance of improving and maintaining high CRF, which can be achieved through a minimum of 150 minutes a week of moderate to vigorous intensity activity, to reduce cancer risk, but this factor does not interact with obesity.

## Methods

### Study design, setting and subjects

The Copenhagen Male Study (CMS) is a prospective cohort study of middle-aged men employed in large private or public workplaces in Copenhagen, Denmark. This study was established to assess the relationship between PA or CRF and coronary heart disease in relatively healthy men. Details of the study design, sampling method, data collection and examination have been published elsewhere^43^. 6,125 eligible men aged between 39 to 61 years were invited to participate; of them, 5,245 provided informed consent to participate in the study and underwent a medical examination, which consisted of a short interview by a physician based on prior completion of a standardized questionnaire, measurement of blood pressure, height, weight and CRF^43^. Recruitment was conducted between 1970 and 1971 and the estimated response rate was 86 per cent. In 1985–1986, 3,260 men completed a questionnaire to update exposures, lifestyle and disease diagnosis. For the purpose of this analysis, we used data collected at baseline (1970–71) and second wave (1985–86) from the CMS study and record linkage data from the Danish Cancer Registry and the Danish Register of Causes of Death. This analysis was approved by the steering committee of the Copenhagen Male Study and was conducted in accordance with relevant guidelines and regulations. The datasets generated during and/or analysed during the current study are not publicly available due to ethical reasons but are available from the corresponding authors on reasonable request.

### Identification of cases

For the individual cancer types examined in this analysis, incident cancers were identified and dates of diagnoses obtained through linkage to data from the Danish Cancer Registry for all cancer registrations until the 22^nd^ of March 2017. This cancer registry is population-based and contains records for all incident malignant neoplasms in the Danish population from 1943 onwards. Although reporting to the cancer registry has been mandatory since 1987, the prior voluntary system ensured completeness and high quality data based on multiple reports from different sources, including hospitals, treatment, follow-up of cancer patients and death certificates^44^. Mortality data were obtained from the Danish Register of Causes of Death, which includes individual data on all deaths among Danish, Greenlanders and Faroese residents dying in Denmark, Greenland or the Faroe Islands^45^. Cancer incidence and mortality were coded to 3 digits using any of the International Classification of Diseases (ICD) 8–10; the following codes were used for PC 185/C61, CRC 153-154.1/C18-20, oral/digestive 140-159/C00-26, respiratory/intrathoracic 160-163/C30-39, genito-urinary 185-189/C60-68, other cancers 170-184,190-209/C40-58,C69-96; and all cancers combined except melanoma and other malignant neoplasms of skin 140-209/C00-96. Skin cancers were excluded as the association with PA might be confounded by ultraviolet (UV) sun exposure and increased risk of sunburn^46^.

### Data collection

Questionnaires collected self-reported information on age, occupation, parental history of coronary heart disease, hypertension, diabetes mellitus, personal medical history and health behaviours, including: PA, daily alcohol intake and smoking habits. SES was derived from Svalastoga’s system which is based on educational attainment and job profile^42^.

### Exposure variables

#### Assessment of body mass index

Body mass index was calculated at baseline and second wave from measured weight and height, dividing weight in kilograms by the square of height in meters (kg/m^2^). Height and weight were ascertained with the subject wearing light clothing and shoes; 2 centimetres were deducted from the height and 2 kilograms from weight^43^. Participants with extreme measures of BMI (<15 kg/m^2^ or >50 kg/m^2^) were excluded from the analysis to reduce the probability of measurement error^47^. All remaining participants were categorised into the recommended BMI categories by WHO; those with a BMI <25 kg/m^2^ (Healthy), ≥25 kg/m^2^ to <30 kg/m^2^ (Overweight) and ≥30 kg/m^2^ (Obese) as time-varying exposures. Missing values of BMI were imputed using the method of Last Observation Carry Forward (LOCF)^48^ since baseline BMI and a re-measurement years later are highly correlated 0.90^22^. Underweight participants (BMI <18.5 kg/m^2^) were combined with the healthy group as this number was too small to influence observed associations (n = 27). The category corresponding to the lowest BMI was used as the reference group.

#### Assessment of physical activity

Physical activity was assessed at baseline and second wave with a closed-ended question in the respective questionnaires as “How much physical activity do you believe you do” *almost nothing* (reference), *some* or *a lot* as time varying exposure. This question was developed by Finn Gyntelberg as there was no reliable or valid PA question at the time of inception or follow-up^43^. The lowest group of PA was used as the reference.

#### Assessment of Cardiorespiratory fitness

Cardiorespiratory fitness was determined only at baseline, using an indirect method of VO_2_ max. This approach relies on heart rate, work load from a bicycle ergometer and the Åstrand nomogram^43^. Heart rate was measured during a submaximal bicycle work in a steady state with the aid of a stethoscope and a stopwatch. The loads used were 100, 150 and 200 watts. One, two or in a few cases three different loads were used. The chosen load for each case was determined from weight and age of the subject or heart rate during the first minute of the test. The examination was supervised by an experienced physician with the assistance of trained nurses^43^. The effect of CRF in statistical analysis was obtained per 10 ml/kg/min increase in VO_2_ max so as to obtain reasonable-sized risk estimates.

#### Confounders

Potential confounders for cancer incidence and mortality included: birth cohort obtained from date of birth (1910s, 1920s or 1930s), smoking status (never, former or current) and grams of tobacco a day (current tobacco smoking was calculated from information about the number of cigarettes, cheroots or cigars, or the weight of pipe tobacco smoked daily. One cigarette was taken as equivalent to 1 g of tobacco, one cheroot as 3 g and one cigar as 4 g), alcohol consumption (2 or less, 3–5, or >6 units a day), diabetes (yes or no), systolic and diastolic blood pressure, history of acute myocardial infarction (AMI) (yes or no) and SES (low, middle or high). Most of the selected confounders were incorporated as time-varying risk factors with the exception of AMI and SES.

### Statistical methods

Analyses were conducted separately for PC, CRC and cancer groupings. Adjusted hazard ratios (HRs) and 95% confidence intervals (CIs) were estimated for cancer incidence and cause-specific mortality, using Cox regression with age as the underlying time scale. Time-varying risk factor analysis was conducted for BMI and self-reported PA and fixed baseline risk factor for CRF. For cancer incidence, participants were censored if they died, were diagnosed with other cancers or were alive at the end of follow up period, whichever came first. For cancer mortality, participants were censored if they died from other causes or were alive at the end of follow-up.

We examined potential two-way interactions between (i) BMI and PA, and (ii) BMI and CRF, on cancer outcomes by adding appropriate interaction terms into the respective models. Based on the nature of the interaction, the multiplicative scale was used as presence of interaction on this scale will also be present on the additive scale^49^. Furthermore, the proportional hazard assumptions of the Cox regression models were assessed by Wald tests of covariates through log-time interactions. Sensitivity analyses were also performed by excluding: (i) underweight (BMI <18.5 kg/m^2^) participants from the lowest BMI category and (ii) the first ten years of follow-up to reduce the potential impact of reverse causality. Statistical analyses were performed using R version 3.4.0.

## Electronic supplementary material

Supplementary material
