# Supplementary material for: Main and interactive effects of physical activity, fitness and body mass in the prevention of cancer from the Copenhagen Male Study
Source: Sci Rep. 2018 Aug 6;8:11780. doi: 10.1038/s41598-018-30280-5 (PMC6078972; doi:10.1038/s41598-018-30280-5)
Supplement: Supplementary file 1 — Supplementary material [file 41598_2018_30280_MOESM1_ESM.pdf]

Supplementary material

Main and interactive effects of physical activity, fitness and body mass in the prevention of cancer from the Copenhagen Male Study.

Authors: Carlos Nunez, Johan Clausen, Magnus Thorsten Jensen, Andreas Holtermann, Finn Gyntelberg, Adrian Bauman.

Supplementary Table S1. Adjusted hazard ratios (HR) and 95% confidence intervals (CI) for cancer incidence according to time-varying BMI and PA; and baseline CRF in the CMS, excluding the first ten years of follow-up and underweight participants.

		*BMI Kg/m^2 HR (95% CI)**				*Physical Activity HR (95% CI)**				*CRF**	
Cancer type	**Events**	**<25**	**≥25-<30**	**≥30**	**P-value[A]**	**Almost nothing**	**Some**	**A lot**	**P-value[A]**	**10 ml/kg/min (VO_2 max)**	**P-value[A]**
Prostate	371	1.00	1.04 (0.83-1.29)	0.76 (0.47-1.23)	0.26	1.00	0.73 (0.53-1.02)	0.77 (0.55-1.07)	0.12	1.00 (0.86-1.16)	0.96
Colorectal	275	1.00	1.12 (0.86-1.45)	0.67 (0.38-1.18)	0.17	1.00	1.12 (0.74-1.69)	1.08 (0.71-1.66)	0.71	0.93 (0.78-1.11)	0.42
Oral and digestive	494	1.00	1.14 (0.94-1.39)	0.98 (0.68-1.41)	0.91	1.00	1.25 (0.92-1.70)	1.07 (0.78-1.47)	0.68	0.90 (0.79-1.03)	0.12
Respiratory and thoracic	388	1.00	0.85 (0.68-1.06)	0.54 (0.34-0.86)	0.01	1.00	0.91 (0.67-1.25)	0.83 (0.60-1.16)	0.27	0.72 (0.61-0.84)	0.001
Genito-urinary	518	1.00	1.07 (0.88-1.29)	0.81 (0.55-1.20)	0.30	1.00	0.76 (0.58-1.00)	0.75 (0.57-0.99)	0.04	1.03 (0.90-1.16)	0.70
Other cancer	311	1.00	1.01 (0.79-1.30)	1.12 (0.72-1.75)	0.61	1.00	1.04 (0.71-1.53)	1.02 (0.69-1.51)	0.92	0.92 (0.78-1.09)	0.35
All-cancers	1,711	1.00	1.02 (0.92-1.13)	0.84 (0.68-1.03)	0.09	1.00	0.97 (0.83-1.13)	0.89 (0.76-1.05)	0.17	0.90 (0.83-0.96)	0.003

*Multivariable model adjusted for: birth decades, smoking and grams of tobacco a day, alcohol, SES, systolic blood pressure, diastolic blood pressure, previous AMI, diabetes and the other study variables. (all covariates are time-dependent except for birth decade, previous AMI, SES and CRF).

[A] 'P-value' for each variable corresponds to a test of whether all HRs = 1

Supplementary Table S2. Adjusted hazard ratios (HR) and 95% confidence intervals (CI) for cancer related death according to time-varying BMI and PA; and baseline CRF in the CMS, excluding the first ten years of follow-up and underweight participants.

		BMI Kg/m² HR (95% CI)				*Physical Activity HR (95% CI)*				*CRF*	
Cancer type	**Events**	**<25**	**≥25-<30**	**≥30**	**P-value**	**Almost nothing**	**Some**	**A lot**	**P-value**	**10 ml/kg/min (VO_2 max)**	**P-value**
Prostate	245	1.00	1.01 (0.77-1.32)	0.66 (0.36-1.21)	0.18	1.00	0.78 (0.51-1.18)	0.84 (0.55-1.29)	0.44	0.96 (0.80-1.16)	0.67
Colorectal	197	1.00	0.98 (0.72-1.32)	0.73 (0.40-1.35)	0.32	1.00	0.87 (0.56-1.37)	0.83 (0.52-1.31)	0.42	0.89 (0.72-1.10)	0.28
Oral and digestive	398	1.00	1.00 (0.80-1.24)	0.94 (0.64-1.39)	0.96	1.00	1.12 (0.81-1.54)	0.85 (0.60-1.20)	0.34	0.89 (0.76-1.03)	0.12
Respiratory and thoracic	431	1.00	0.90 (0.73-1.11)	0.49 (0.30-0.78)	0.003	1.00	0.92 (0.68-1.24)	0.80 (0.58-1.10)	0.17	0.66 (0.58-0.79)	<0.001
Genito-urinary	364	1.00	0.95 (0.76-1.19)	0.66 (0.41-1.08)	0.10	1.00	0.96 (0.67-1.37)	0.99 (0.69-1.43)	0.95	0.92 (0.79-1.08)	0.31
Other cancer	299	1.00	0.76 (0.59-0.98)	0.77 (0.48-1.23)	0.04	1.00	1.20 (0.80-1.80)	1.13 (0.74-1.72)	0.56	0.87 (0.73-1.03)	0.11
All-cancers	1,489	1.00	0.92 (0.82-1.03)	0.74 (0.59-0.92)	0.006	1.00	0.99 (0.84-1.17)	0.90 (0.76-1.07)	0.24	0.82 (0.76-0.89)	<0.001

*Multivariable model adjusted for: birth decades, smoking and grams of tobacco a day, alcohol, SES, systolic blood pressure, diastolic blood pressure, previous AMI, diabetes and the other study variables. (all covariates are time-dependent except for birth decade, previous AMI, SES and CRF).

A ‘P-value’ for each variable corresponds to a test of whether all HRs = 1

Supplementary Figure S1. Adjusted hazard ratios and 95% CI for the interaction between BMI and PA on cancer incidence of All-cancers combined, colorectal and prostate cancer.

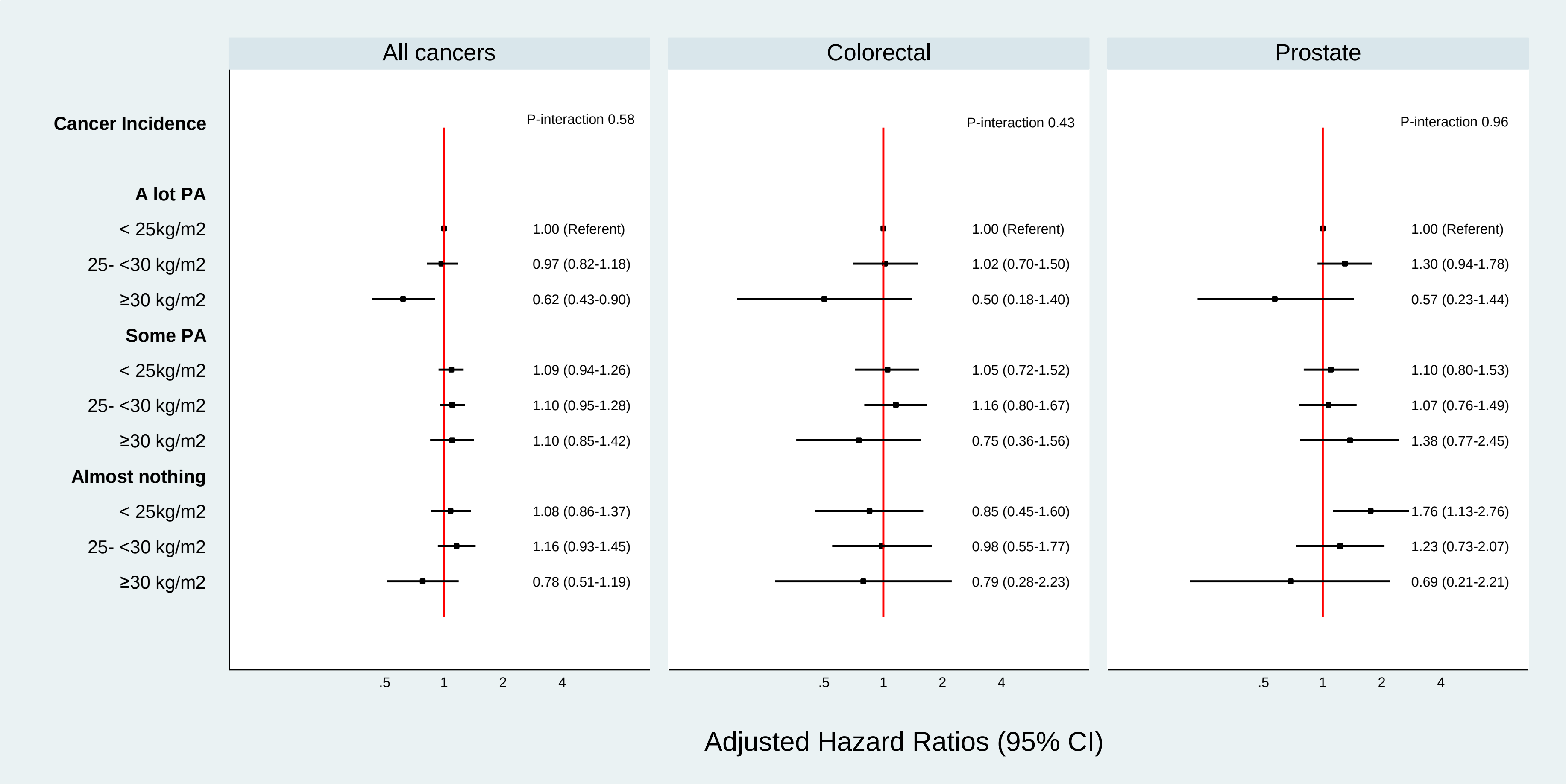

*Multivariable model adjusted for: birth decades, smoking and grams of tobacco a day, alcohol, SES, systolic blood pressure, diastolic blood pressure, previous AMI, diabetes, physical activity, BMI, CRF and interaction between BMI-PA.

Supplementary Figure S2. Adjusted hazard ratios and 95% CI for the interaction between BMI and PA on cancer incidence of genito-urinary, oral and digestive, other cancers; and respiratory and thoracic cancers.

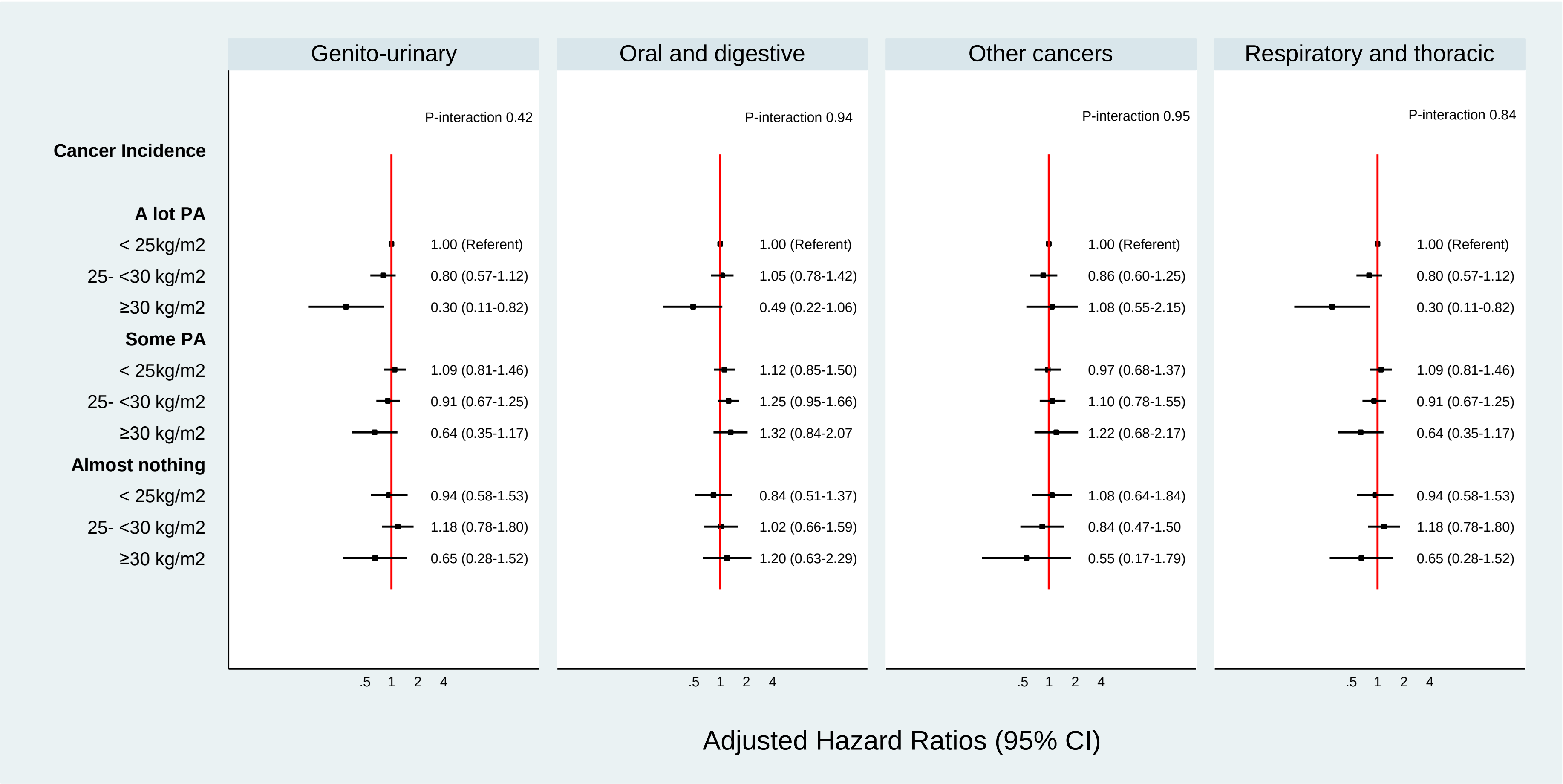

*Multivariable model adjusted for: birth decades, smoking and grams of tobacco a day, alcohol, SES, systolic blood pressure, diastolic blood pressure, previous AMI, diabetes, physical activity, BMI, CRF and interaction between BMI-PA.

Supplementary Figure S3. Adjusted hazard ratios and 95% CI for the interaction between BMI and PA on cancer mortality of All-cancers combined, colorectal and prostate cancer.

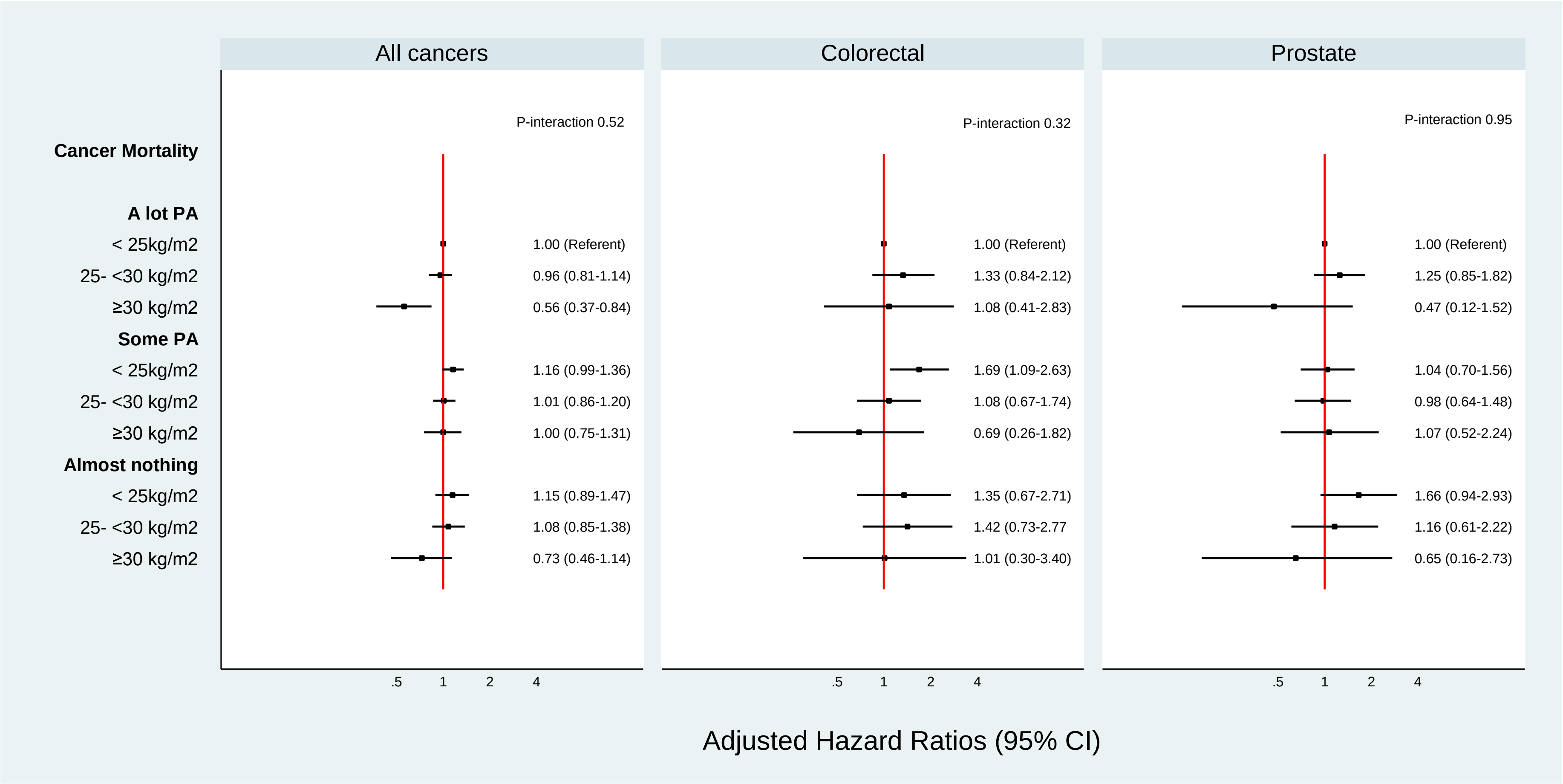

*Multivariable model adjusted for: birth decades, smoking and grams of tobacco a day, alcohol, SES, systolic blood pressure, diastolic blood pressure, previous AMI, diabetes, physical activity, BMI, CRF and interaction between BMI-PA.

Supplementary Figure S4. Adjusted hazard ratios and 95% CI for the interaction between BMI and PA on cancer mortality of genito-urinary, oral and digestive, other cancers; and respiratory and thoracic cancers.

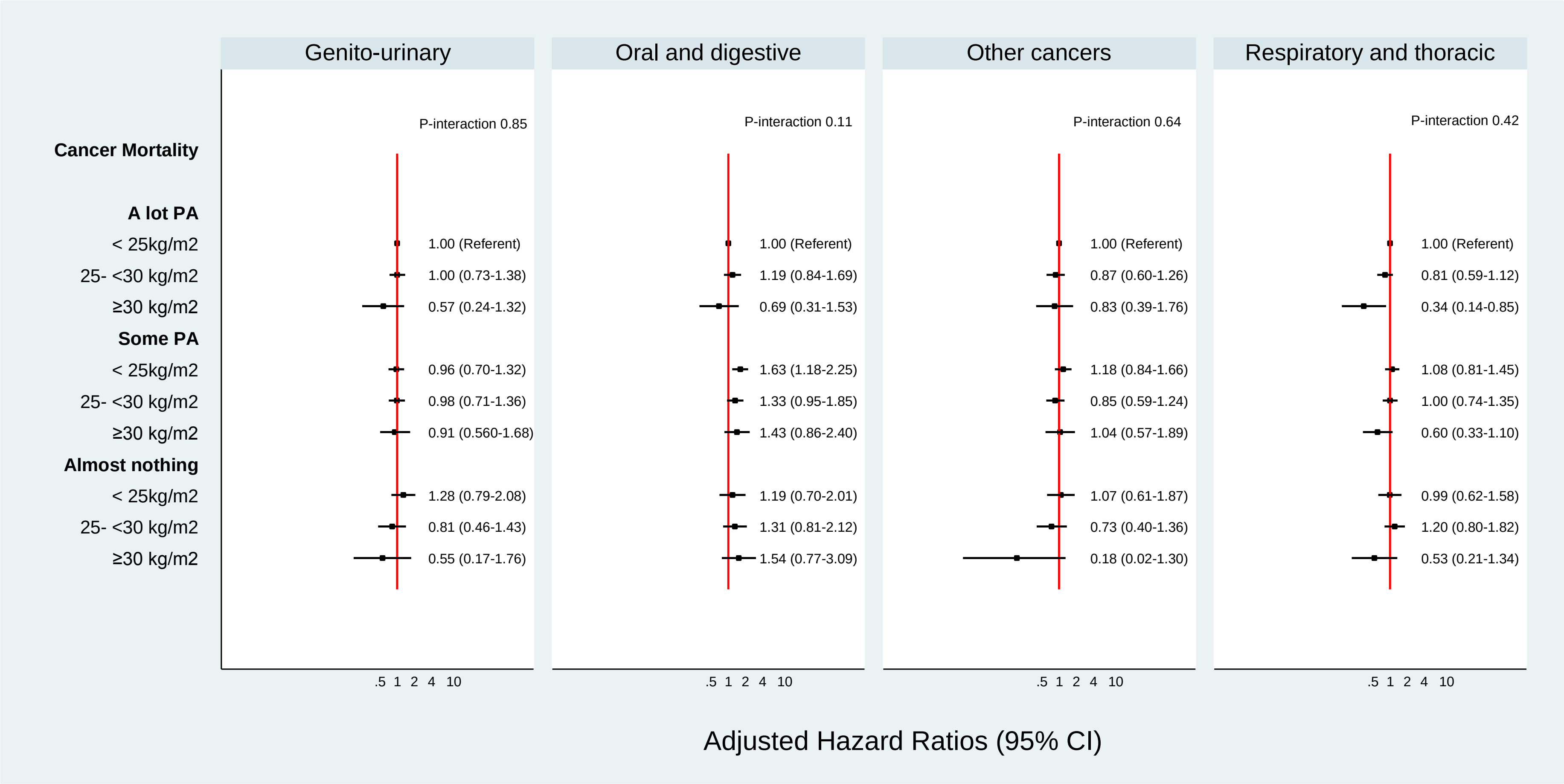

*Multivariable model adjusted for: birth decades, smoking and grams of tobacco a day, alcohol, SES, systolic blood pressure, diastolic blood pressure, previous AMI, diabetes, physical activity, BMI, CRF and interaction between BMI-PA.
